# Impact of Continuous Renal Replacement Therapy Initiation on Urine Output and Fluid Balance: A Multicenter Study

**DOI:** 10.1159/000530146

**Published:** 2023-04-18

**Authors:** Kyle C. White, Kevin B. Laupland, Emily See, Ary Serpa-Neto, Rinaldo Bellomo

**Affiliations:** a Intensive Care Unit, Princess Alexandra Hospital, Woolloongabba, QLD, Australia; b Faculty of Medicine, University of Queensland, Brisbane, QLD, Australia; c Department of Intensive Care Services, Royal Brisbane and Women’s Hospital, Brisbane, QLD, Australia; d Queensland University of Technology (QUT), Brisbane, QLD, Australia; e School of Medicine, University of Melbourne, Melbourne, VIC, Australia; f Department of Critical Care, University of Melbourne, Melbourne, VIC, Australia; g Department of Intensive Care, Austin Hospital, Heidelberg, VIC, Australia; h Department of Nephrology, The Royal Melbourne Hospital, Parkville, VIC, Australia; i Department of Nephrology, The Royal Children’s Hospital, Parkville, VIC, Australia; j Australian and New Zealand Intensive Care Research Centre (ANZIC-RC), School of Public Health and Preventive Medicine, Monash University, Melbourne, VIC, Australia; k Department of Critical Care Medicine, Hospital Israelita Albert Einstein, São Paulo, Brazil

**Keywords:** Critical care, Acute kidney injury, Continuous renal replacement therapy

## Abstract

**Introduction:**

The effect of continuous renal replacement therapy (CRRT) on renal function is poorly understood. However, the initiation of CRRT may induce oliguria. We aimed to investigate the impact of CRRT commencement on urine output (UO).

**Methods:**

This was a retrospective cohort study in two intensive care units. We included all patients who underwent CRRT and collected data on hourly UO and fluid balance before and after CRRT commencement. We performed an interrupted time series analysis using segmented regression to assess the relationship between CRRT commencement and UO.

**Results:**

We studied 1,057 patients. Median age was 60.7 years (interquartile range [IQR], 48.3–70.6), and the median APACHE III was 95 (IQR, 76–115). Median time to CRRT was 17 h (IQR, 5–49). With start of CRRT, the absolute difference in mean hourly UO and mean hourly fluid balance was −27.0 mL/h (95% CI: −32.1 to −21.8; *p* value < 0.01) and – 129.3 mL/h (95% CI: −169.2 to −133.3), respectively. When controlling for pre-CRRT temporal trends and patient characteristics, there was a rapid post-initiation decrease in UO (−0.12 mL/kg/h; 95% CI: −0.17 to −0.08; *p* value < 0.01) and fluid balance (−78.1 mL/h; 95% CI: −87.9 to −68.3; *p* value < 0.01), which was sustained over the first 24 h of CRRT. Change in UO and fluid balance were only weakly correlated (*r* −0.29; 95% CI: −0.35 to −0.23; *p* value < 0.01).

**Conclusion:**

Commencement of CRRT was associated with a significant decrease in UO that could not be explained by extracorporeal fluid removal.

## Introduction

Continuous renal replacement therapy (CRRT) is administered to approximately 5% of patients admitted to intensive care units (ICUs) [1, 2]. Individuals who receive CRRT in the ICU have a high mortality, with rates ranging from 30 to 70% [2, 3]. Of the survivors, 10–30% will require dialysis long term [2, 4].

Despite extensive use of CRRT in the critically ill, its impact on renal function remains uncertain [3]. There are concerns of possible harm induced by performing early renal replacement therapy (RRT) [2]. Several mechanisms have been proposed to cause such RRT-associated kidney injury and include induction or exacerbation of hypotension, vascular access-related complications, myocardial injury, and volume depletion associated with a rapidly induced negative fluid balance with associated decrease in urine output (UO).

UO is a universal real-time marker of kidney function [5] and is a key component of the acute kidney injury definition [6]. Previous research has identified increasing UO during CRRT as a predictor of successful discontinuation, demonstrating the importance of residual renal function in critically ill patients on CRRT [7, 8]. However, the impact of CRRT commencement on UO has not been previously investigated. The objective of this study was to investigate the effect of initiation of CRRT on UO and examine how this may relate to fluid balance shifts among patients admitted to ICU.

## Methods

### Study Design

This was a multicenter, retrospective cohort study, utilizing granular routinely collected clinical data.

### Study Sites and Participants

The study sites were two closed-model tertiary ICUs located in metropolitan Brisbane, Queensland, Australia: The Royal Brisbane and Women’s Hospital (RBWH) and the Princess Alexandra Hospital (PAH). Both centers are large academic institutions that are the statewide referral centers for neurosurgical and trauma patients. The RBWH has a total of 33 ICU beds and is also the state burns center. The PAH has a total of 30 ICU beds, includes cardiothoracic patients, and is the statewide referral center for liver transplant and spinal injury patients.

We evaluated all adult patients admitted to the participating ICUs who received CRRT between January 1, 2015, and December 31, 2020. Patients were eligible if their records included UO data for at least 4 h prior to CRRT and for 24 h after CRRT commencement. We excluded patients with chronic kidney disease requiring chronic dialysis and readmission episodes within the same hospital admission.

### Data Sources

Routinely collected data were obtained from both centers using the eCritical MetaVision^™^ (iMDsoft, Boston, MA, USA) clinical information systems. Baseline demographics, daily laboratory data, hourly fluid balance, and CRRT data were obtained. Information on admission diagnosis and severity of illness was based on the Acute Physiology and Chronic Health Evaluation III (APACHE III) score and coded and extracted according to the Australia and Zealand Intensive Care Society (ANZICS) Centre for Outcome and Resource Evaluation (CORE) Adult Patient Database (APD) data dictionary. The noradrenaline equivalent score [9] and the vasoactive inotrope score [10] were derived based on previously described methods.

### Outcomes

The primary outcome was UO, in mL/kg. The secondary outcome was fluid balance, in mL.

### Statistical Analysis

Continuous data are presented as medians (interquartile range [IQR]) or means with standard deviations (SDs) and categorical variables as *n* (%). An interrupted time series analysis using segmented regression was performed to test the relationship between CRRT commencement and outcomes. We performed the analysis separately for each outcome variable described above. The pre- and post-CRRT periods were defined as the 24 h before and after CRRT commencement, respectively. All models were fit using linear regression with robust standard errors clustered at the hospital level. All models controlled for the following patient characteristics: age, gender, body mass index, APACHE III, and vasopressor-inotropic score (VIS) on day 1 [10]. A Q-Q plot, shown in online supplementary eFigure 1 (for all online suppl. material, see www.karger.com/doi/10.1159/000530146), of UO measurements demonstrated minimal extreme values.

To test the association between the CRRT commencement and outcomes, a segmented regression approach was used (full equation model described in the Online Supplement). In the model, the continuous time variable was included to control for pre-CRRT temporal trends. This approach accounts for the fact that UO and fluid balance were generally changing before CRRT. In addition, the start of CRRT was included to assess the change immediately after start of CRRT. Finally, the post-CRRT time was included to estimate the change in the trend between post- and pre-CRRT periods. The primary test of the association between start of CRRT and patient outcomes was a joint test that either the immediate change or the change in slope was equal to zero.

All models were performed at the patient level considering the patients as random effects to account for repeated measurement but were presented graphically after aggregation per period to facilitate visualizing the trends over time. A sensitivity analysis excluding patients who received furosemide before or after CRRT commencement was performed. To test for an association between change in UO and fluid balance with the commencement of CRRT, a Pearson correlation coefficient was performed.

Statistical analyses were performed using R v.4.0.3. All tests were 2-sided, and for other comparisons, *p* value of 0.05 or less was considered significant.

## Results

### Patients

From January 1, 2015, to December 31, 2020, 1,267 patients received CRRT and were eligible for inclusion. After the exclusion of 167 (13.2%) patients with end-stage kidney disease and 43 (3.9%) readmissions, 1,057 (83.4%) patients were included.

The patient characteristics are shown in Table 1. The median age was 60.7 (IQR, 48.3–70.6) years, 683 (64.6%) were male, and the median body mass index was 26.3 (IQR, 23.7–30.8). The severity of illness as measured by the median APACHE III was 95 (IQR, 76–115), and the most common source of admission was the emergency department. The most common diagnostic categories were sepsis (263, 25.0%) and cardiovascular conditions (248, 23.6%).

**Table 1.** Baseline characteristics of the included patients

	Overall (*n* = 1,057)
Age, years	60.7 (48.3–70.6)
Male gender, *n* (%)	683 (64.6)
BMI, kg/m^2^	26.3 (23.7–30.8)
APACHE III	95 (76–115)
SOFA on day 1	10 (7–12)
Respiratory	2 (0–3)
Cardiovascular	3 (1–4)
Renal	4 (3–4)
Elective admission, *n* (%)	62 (5.9)
ICU source of admission, *n* (%)	
Emergency department	372 (35.2)
Operating room	261 (24.7)
Other hospital	57 (5.4)
Other ICU	99 (9.4)
Ward	268 (25.4)
Admission category, *n* (%)	
Cardiovascular	248 (23.6)
Gastrointestinal	184 (17.5)
Hematological	20 (1.9)
Musculoskeletal or skin	37 (3.5)
Neurological	27 (2.6)
Renal or genitourinary	68 (6.5)
Respiratory	66 (6.3)
Sepsis	263 (25.0)
Trauma	138 (13.1)
Other	2 (0.2)
Organ support on day 1	
Invasive ventilation, *n* (%)	610 (78.6)
Vasopressor, *n* (%)	564 (53.4)
Noradrenaline dose, μg/min	10.2 (5.3–19.1)
Noradrenaline equivalence score	0.0 (0.0–0.2)
Vasoactive-inotrope score	3.7 (0.0–16.1)
CRRT characteristics	
Hours until start of CRRT	17 (5–49)
CRRT mode, *n* (%)	
CVVHF	36 (3.5)
CVVHD	2 (0.2)
CVVHDF	989 (96.3)
CRRT anticoagulation, *n* (%)	
Citrate	487 (47.4)
Heparin	198 (19.3)
Other/none	343 (33.4)
Duration of therapy, h	67 (29–169)
In survivors	75 (37–185)
Arterial blood gas on day 1	
pH	7.28 (7.19–7.35)
PaO_2_/FiO_2_	186 (123–267)
Base excess, mmol/L	−8 (−12–−4)
Bicarbonate, mmol/L	19 (16–22)
Lactate, mmol/L	2.4 (1.5–5.3)
Pathology on day 1	
Highest creatinine, μmol/L	279 (176–414)
Highest urea, mmol/L	17.7 (10.6–26.7)
Highest potassium, mmol/L	4.7 (4.3–5.3)
Clinical outcomes	
ICU LOS, days	7 (3–14)
In survivors	6 (3–12)
Hospital LOS, days	21 (9–42)
In survivors	23 (15–45)
ICU mortality, *n* (%)	319 (30.7)
Hospital mortality, *n* (%)	407 (50.8)

Data are median (quartile 25%–quartile 75%) or *n* (%). Percentages may not total 100 because of rounding.

APACHE, Acute Physiology and Chronic Health Evaluation; SOFA, Sequential Organ Failure Assessment; ICU, intensive care unit; CRRT, continuous renal replacement therapy; CVVHF, continuous veno-venous hemofiltration; CVVHD, continuous veno-venous hemodialysis; CVVHDF, continuous veno-venous hemodiafiltration; NUF, net ultrafiltration; BMI, body mass index; LOS, length of stay.

### Intensive Care Therapies and Outcomes

On day 1, 610 (78.6%) study patients were ventilated with a median PaO_2_/FiO_2_ ratio of 186 (IQR, 123–267). Furthermore, most patients were receiving vasopressors (564, 53.4%) with a median VIS of 3.7 (IQR, 0.0–16.1). In the 24 h prior to CRRT commencement, 180 (17.0%) patients received furosemide with a median dose of 15.8 (IQR, 10.0–20.0) mg/h. The treatment characteristics in the 24 h before and after CRRT are shown in online supplementary eTable 1. The median hospital length of stay was 21 (IQR, 9–42) days, and mortality was 30.7% and 50.8% for ICU and hospital, respectively.

### CRRT Characteristics

The median time from admission until the first CRRT session was 17 (IQR, 5–49) hours, and the median duration of CRRT was 67 (IQR, 29–169) hours. Almost all patients received hemodiafiltration (989, 96.3%) with regional citrate being the most prescribed anticoagulation technique (487, 47.4%). The median hourly fluid removal was 33.6 (IQR, 5.0–102.7) mL/h. During CRRT, the use of invasive ventilation was 83.6% and vasopressors were used in 85.1%. The CRRT characteristics are shown in online supplementary eTable 2. In the 24-h post CRRT, 36 (3.4%) patients received furosemide with a median hourly dose of 10.0 (IQR, 7.3–20.0) mg/h.

#### Urine Output

The UO values before and after CRRT are reported in online supplementary eTable3. In the 24 h prior to CRRT, the mean hourly UO was 60.2 (SD ± 142.8) mL or 0.75 (SD ± 1.78) mL/kg/h. In patients who received furosemide, the mean hourly UO was 58.2 (SD ± 133) mL or 0.7 (SD ± 1.8) mL/kg/h. In the 24 h after CRRT, the mean hourly UO was 34.3 (SD ± 108.8) mL/h or 0.4 (SD ± 1.4) mL/kg/h. The absolute difference in mean hourly UO before and after CRRT was −27.0 mL/h (95% CI: −32.1 to −21.8; *p* value < 0.01).

**Fig. 1.**
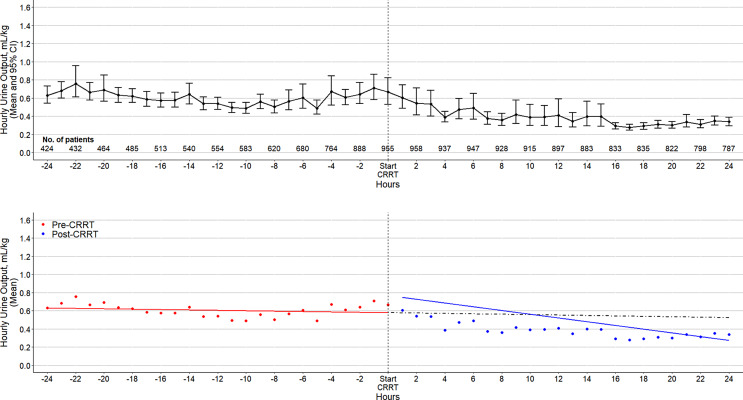
Cumulative UO and fluid balance over time. Top: cumulative UO over time in all patients. Circles are mean, and error bars, 95% confidence intervals. Bottom: cumulative fluid balance over time in all patients. Circles are mean, and error bars, 95% confidence intervals.

The results of the primary outcome analysis appear in Table 2. Controlling for patient characteristics and pre-CRRT temporal trends, the start of CRRT was associated with a rapid drop in UO (−0.122 mL/kg/h; 95% CI: −0.165 to −0.079; *p* value < 0.01). Furthermore, the UO remained depressed over the 24 h after CRRT (−0.011 mL/kg/h; 95% CI: −0.015 to −0.009; *p* value < 0.01). A graphical representation is shown in Figure 1 and in online supplementary eFigure 2. A sensitivity analysis excluding patients who received furosemide before or after CRRT showed no material change to the findings as shown in online supplementary eTable 4.

**Table 2.** Adjusted estimates of the association between CRRT commencement and patient outcomes from the analysis for the primary and secondary outcomes

	Urine output, mL/kg/h		Fluid balance, mL/h	
	mean difference^a,b^ (95% CI)	*p* value	mean difference^a,b^ (95% CI)	*p* value
Pre-CRRT	−0.001 (−0.003–0.002)	0.91	−0.456 (−1.035–0.126)	0.12
Immediate change after CRRT	−0.122 (−0.165 to −0.079)	<0.01	−78.090 (−87.870 to −68.305)	<0.01
Change in slope after CRRT	−0.011 (−0.015 to −0.009)	<0.01	−2.228 (−2.982 to −1.476)	<0.01

CI, confidence interval; CRRT, continuous renal replacement therapy; BMI, body mass index.

^a^All models were fit using linear regression with robust standard errors clustered at the hospital level. All models controlled for the following patient characteristics: age, gender, BMI, APACHE III, and VIS on day 1. All models adjusted for pre-CRRT temporal trends and performed on the patient level considering the patients as random effects to account for repeated measurement.

^b^The primary test of the association between the CRRT commencement and the patient outcomes was a joint test that either the immediate change or the change in slope was equal to zero.

### Fluid Balance

**Fig. 2.**
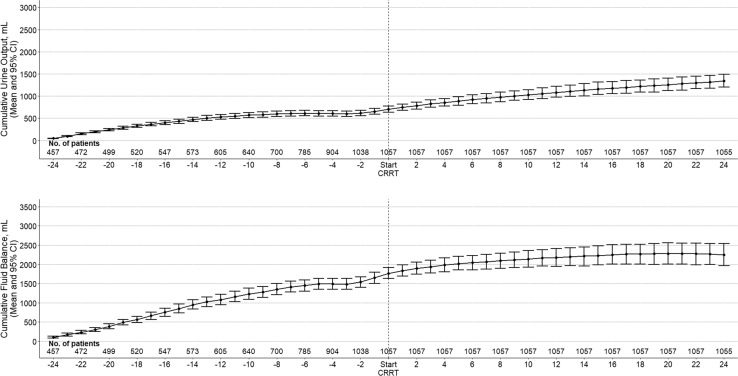
UO over time in all patients. Top: UO over time in all patients. Circles are mean, and error bars, 95% confidence intervals. Bottom: circles are the mean UO in each time point. Solid lines represent fitted lines from the segmented regression, and dashed line is the counterfactual outcome had the pre-CRRT trends continued.

Fluid balance before and after CRRT is reported in online supplementary eTable 1. The fluid balance in the 24 h prior to CRRT commencement was 1,403 (IQR, 233–2,765) mL with a mean hourly fluid balance of +150 (SD ± 329) mL/h. In patients who received furosemide, the mean hourly fluid balance was +173 (SD ± 371) mL/h. The absolute difference in mean hourly fluid balance before and after CRRT was – 129 mL/h (95% CI: −169 to −133; *p* value < 0.01). After commencement of CRRT, the mean hourly fluid balance became negative only at 22 h, and at 24 h, the cumulative fluid balance remained positive at 2,250 mL, as shown in Figure 2.

**Fig. 3.**
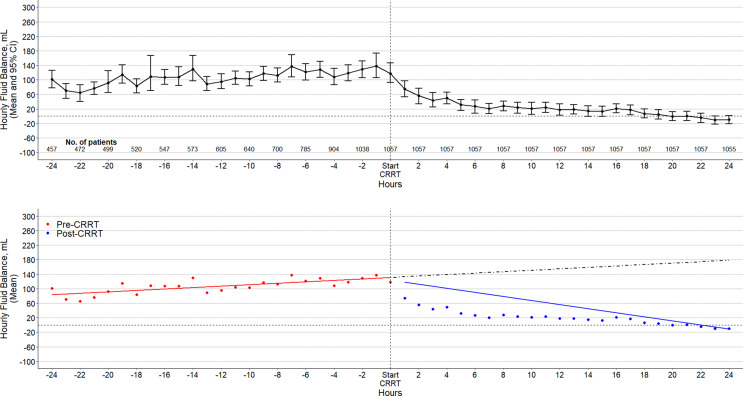
Fluid balance over time in all patients. Top: fluid balance over time in all patients. Circles are mean, and error bars, 95% confidence intervals. Bottom: circles are the mean fluid balance in each time point. Solid lines represent fitted lines from the segmented regression, and the dashed line is the counterfactual outcome had the pre-CRRT trends continued.

After controlling for patient characteristics and pre-CRRT temporal trends, the start of CRRT was associated with a rapid decrease in hourly fluid balance (−78 mL/h; 95% CI: −88 to −68; *p* value < 0.01), which, despite reduction, remained positive as demonstrated in Figure 3. The fluid balance trend over time is shown in Figure 3 and in online supplementary eFigure 2. The sensitivity analysis considering furosemide usage did not alter the outcomes of the analysis, as demonstrated in online supplementary eTable 4.

### Association between UO and Fluid Balance

**Fig. 4.**
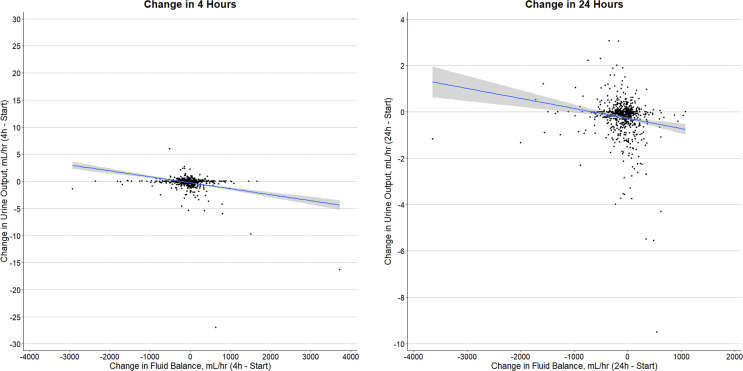
XY plot of change in urine output versus change in fluid balance. Left: change in hourly UO from 0 h to 4 h on the Y axis compared to change in hourly fluid balance from 0 h to 4 h on the X axis. Right: change in hourly UO from 0 h to 24 h on the Y axis compared to change in hourly fluid balance from 0 h to 24 h on the X axis.

We assessed for an association between changes in fluid balance and UO by performing a Pearson correlation test. UO and fluid balance were only weakly associated (*r* −0.29; 95% CI: −0.35 to −0.23; *p* value < 0.01). As shown in Figure 4**,** the XY plot suggests that the reduction in mean hourly UO after commencement of CRRT was associated with more positive mean hourly fluid balance. Furthermore, given the potential influence of patients with oliguria on this analysis, we assessed only non-oliguric patients (*r* −0.44; 95% CI: −0.54 to −0.34; *p* value < 0.01) as shown in online supplementary eFigure 3, with comparable results.

### Oliguria Post-CRRT Commencement

We assessed for differences between patients who were oliguric post-CRRT commencement, defined as UO <0.5 mL/kg/h, and those who were not oliguric. We found 806 (78.6%) of the cohort were oliguric post-CRRT commencement. Oliguric patients had a significantly higher NAE score (0.07 vs. 0.00; *p* value < 0.01), had no difference in invasive ventilation requirement (79.7% vs. 75.3%, *p* value 0.23), and had A significantly higher net ultrafiltration rate (0.73 mL/kg/h vs. 0.09 mL/kg/h; *p* value < 0.01).

## Discussion

### Key Findings

In this multicenter study of >1,000 critically ill patients undergoing CRRT, we used hourly data to investigate the relationship between CRRT commencement and UO with additional information on fluid balance. We found that CRRT initiation was associated with a rapid and sustained decrease in UO, even after controlling for pre-CRRT UO trends, use of furosemide, and patient characteristics. Additionally, we found that the pre-CRRT trend of fluid accumulation was not immediately reversed; however, there was a reduction in the mean hourly fluid balance after CRRT to a less positive value. Overall, a negative hourly fluid balance was not achieved until 22 h after instigation of CRRT, and changes in fluid balance were not correlated with UO.

### Relationship to the Literature

Prior to our investigation, there were no known studies that assessed the impact of commencement of CRRT on hourly UO. A post hoc analysis of the ATN trial assessed the impact of RRT intensity on daily UO and demonstrated that UO increased in the less-intensive group and decreased in the more-intensive group [11]. The use of daily UO limits the study’s assessment of the impact of CRRT in the hours after commencement and introduces confounders to the assessment of UO. In addition, the study used multiple RRT modalities other than CRRT, such as intermittent hemodialysis (IHD) and slow low-efficiency dialysis, both of which limit the applicability in the assessment of the impacts of CRRT.

Animal models have demonstrated a reduction in UO with CRRT [12], though the appropriateness of extrapolating these data to the adult human population is uncertain. Similarly, studies in outpatients commencing IHD for end-stage kidney disease have demonstrated a loss of residual renal function as measured by UO [13, 14] with initiation of RRT. The association appears to be dose-dependent [15]. However, these data cannot be extrapolated to critically ill patients receiving undergoing CRRT, given the obvious discrepancies between study populations in terms of the severity of illness, the differences in the underlying kidney injury, acute versus chronic phenotypes, and the stark contrast in dialysis modalities [3].

### Implications of the Study Findings

In a population of critically ill patients, our study suggests that the commencement of CRRT has a negative impact on UO. The magnitude decrement in UO with commencement of CRRT was statistically significant, represents an almost 50% decrease, and occurred rapidly, despite a despite an hourly fluid balance that remained positive. Given that UO is a universally accepted marker of renal function in individuals with acute kidney injury, it is plausible that the decrease in UO on CRRT represents a signal of harm to the injured kidney. The possibility that RRT can harm the kidney is a growing concern with the recent literature demonstrating that the earlier commencement of RRT can increase long-term RRT dependence [2]. This is further supported by a post hoc analysis of the ATN trial that demonstrated less renal recovery in the intensive RRT group [16]. Our study exclusively included patients undergoing CRRT; however, there remains uncertainty if different RRT modalities in the acute setting, such as CRRT and IHD, have similar risk of RRT dependence.

Notably, the hourly fluid balance on CRRT became immediately less positive after the start of CRRT, and the change was similar in direction and magnitude to the change seen in UO trends. In contrast to these similarities, hourly UO and hourly fluid balance demonstrated a negative correlation, such that more positive hourly fluid balance was associated with a greater reduction in hourly UO. The significance of this result is uncertain as the correlation was weak to moderate and the regression curves suggest the effect may have been driven by outliers. As such, the mechanism of the demonstrated reduction in UO immediately after the commencement of CRRT remains unexplained and validates need for further investigation. Future investigation for an association between previously proposed mechanisms, such as myocardial stunning [17, 18], altered hemodynamics [19, 20], and renal hypoperfusion [21], and UO change after CRRT commencement warrants attention.

### Strengths and Limitations

Our study was a large cohort that included a wide array of ICU admissions as these ICUs encompass the full range of adult critical care services except for acute heart and lung transplants. Moreover, our study details the most comprehensive dataset of critically ill patients receiving CRRT with highly granular data, which was electronically extracted from a ubiquitous clinical information system. The data collected were predominantly validated, complete, and given its collection by non-research staff represents a non-biased sample. Adding to the robustness of our study, we conducted a sensitivity analysis on furosemide use to determine its influence on UO, which did not alter the original findings of the study. Furthermore, the study population was remarkably similar to several recent large multinational CRRT trials with similar APACHE III scores, ventilation rates, vasopressor use, and hospital mortality rate [2, 22].

We acknowledge some limitations. Given the retrospective, observational nature of the data, the association demonstrated between UO and CRRT does not imply causality. However, the acquisition 24 h of pre-CRRT data in over 1,000 patients strengthens the suggestions of a direct relationship. The study cohort was entirely from two resource intensive hospitals, which may limit the study application to less resource intensive environments. Yet the cohort examined was large, consisting of 1,057 patients, extracted from two large tertiary academic institutions with a wide breadth of pathology, allowing a degree of external validity to our study. The heterogeneity of the study population with unknown medical comorbidities and a wide range of admission diagnoses potentially modified the outcome analysis. Further exploration of subgroups within the study population will be required to assess for within population differences in UO after CRRT commencement. CRRT indication and hemodynamic variables, such as cardiac output and central venous pressure, were not collected. These data points could interact with the demonstrated association; however, this is somewhat mitigated by the large, heterogenous patient population. Furthermore, baseline weight was not available; therefore, fluid balance as a proportion of body weight could not be calculated, which would be a more accurate measure of fluid balance. Lastly, the interrupted time series analysis assumes an immediate change in the outcome or a change in slope of the outcome and does not account for more complex temporal relationship between CRRT commencement and outcomes.

## Conclusion

Commencement of CRRT in critically ill patients resulted in a statistically significant, near 50% rapid decrease in UO. There was a simultaneous reduction in fluid balance to a less positive value. There was a demonstrable weak association between change in UO and change in fluid balance. Overall, these observations imply a potentially harmful effect on renal function induced by CRRT that is not mediated by changes in fluid balance and suggest the presence of additional mechanisms which might be responsible for the decrease in UO which justifies further systematic investigations.

## Acknowledgments

We thank Rod Hurford and Nermin Karamujic, Informatics and Clinical Information Systems, Princess Alexandra Hospital and Royal Brisbane and Women’s Hospital Intensive Care Units, respectively, for providing study data.

## Statement of Ethics

This study was approved by the Metro South Hospital and Health Service Human Research Ethics Committee (LNR/2020/QMS/68626) with an individual waiver of consent, and therefore, written consent was not required or granted.

## Conflict of Interest Statement

The authors have no conflicts of interest to declare.

## Funding Sources

This research did not receive any specific grant from funding agencies in the public, commercial, or not-for-profit sectors.

## Author Contributions

Study conception and design: all the authors; data acquisition and article drafting: Kyle White; analysis: Ary Serpa-Neto and Kyle White; interpretation of data: Kyle White, Ary Serpa-Neto, Kevin Laupland, Emily See, and Rinaldo Bellomo; article revision for important intellectual content: Kyle White, Ary Serpa-Neto, Kevin Laupland, Emily See, and Rinaldo Bellomo; final approval of the version submitted for publication: Kyle White, Ary Serpa-Neto, Kevin Laupland, Emily See, and Rinaldo Bellomo; agreement to be accountable for all aspects of the work in ensuring that questions related to the accuracy or integrity of any part of the work are appropriately investigated and resolved: Kyle White and Rinaldo Bellomo.

## Data Availability

All data generated or analyzed during this study are included in this published article and its online supplementary material files. Further inquiries can be directed to the corresponding author.
