# Supplementary material for: Impact of Continuous Renal Replacement Therapy Initiation on Urine Output and Fluid Balance: A Multicenter Study
Source: Blood Purif. 2023 Apr 18;52(6):532–40. doi: 10.1159/000530146 (PMC11226201; doi:10.1159/000530146)

ONLINE SUPPLEMENT

**METHODS**

*Description of the model*

To test the association between the CRRT commencement and outcomes, a basic segmented regression was used (Eq. 1). In the model, the continuous time variable was included to control for pre-CRRT temporal trends. This approach accounts for the fact that urine output and fluid balance were generally changing previous to CRRT. In addition, the start of CRRT was included to assess the change immediately after start of CRRT. Finally, the post-CRRT time was included to estimate the change in the trend between post– and pre–CRRT periods. The primary test of the association between start of CRRT and patient outcomes was a joint test that either the immediate change or the change in slope were equal to zero.

$$Y_{T}= \beta_{0}+\beta_{1}*{time}_{t}+\beta_{2}*{CRRT}_{t}+$$

$$\beta_{3}*{time\_post}_{t}+\ldots+\beta_{4}X_{t4}\ldots\beta_{p}X_{tp}+ \varepsilon_{t} (Eq. 1)$$

Where:

1. *Y_t_* is the outcome for time *t*;
2. time is the value of time (from 1 to k) from start of pre-CRRT to end of post-CRRT, and equals *t*;
3. CRRT = 1 for CRRT received and 0 if not received at time *t*;
4. time_post equals 0 if pre-CRRT and otherwise equals number of time periods from start of CRRT to current time period;
5. X are the (*p* – 3) patient-level covariates at time period *t*;
6. ε is a patient-level error term;
7. β_0_ is the baseline level of the outcome at time 0;
8. β_1_ is the slope in the pre-CRRT period;
9. β_2_ is the change in the mean of the outcome just after start of CRRT;
10. β_3_ is the difference in slopes between post- and pre-CRRT periods (i.e., post − pre).

As the primary test of the association between the NUF groups and the patient outcomes was a joint test that either the immediate change or the change in slope were equal to zero. All coefficients were modelled as fixed effects. To account for the correlation of outcomes within a hospital, and for the repeated measurements within a patient, the hospital and the patients were included as random effects.

| **eTable 1 - Clinical Characteristics in the 24 Hours Before and the 24 Hours After CRRT in the Included Patients** | | | |
| --- | --- | --- | --- |
|  | **24 hours Pre-CRRT** | **24 hours Post-CRRT** | ***p* value** |
| Use of frusemide - no. (%) | 180 (17.0) | 36 (3.4) | < 0.01 |
| Cumulative dose, mg | 221 (117 - 347) | 42 (20 - 87) | < 0.01 |
| Per weight, mg/kg | 2.6 (1.2 - 4.3) | 0.5 (0.3 - 1.2) | < 0.01 |
| Mean hourly dose, mg/h | 15.8 (10.0 - 20.0) | 10.0 (7.3 - 20.0) | 0.36 |
| Per weight, mg/kg/h | 0.2 (0.1 - 0.2) | 0.1 (0.1 - 0.2) | 0.22 |
| Urine output |  |  |  |
| Cumulative, mL | 273 (67 - 820) | 222 (49 - 769) | 0.17 |
| Per weight, mL/kg | 3.2 (0.8 - 9.8) | 2.6 (0.5 - 8.8) | 0.16 |
| Mean hourly, mL | 25.2 (8.1 - 62.8) | 11.8 (3.0 - 35.7) | < 0.01 |
| Per weight, mL/kg/h | 0.3 (0.1 - 0.7) | 0.1 (0.0 - 0.4) | < 0.01 |
| Fluid intake |  |  |  |
| Cumulative, mL | 2578 (1164 - 4162) | 3060 (2189 - 4138) | < 0.01 |
| Mean hourly, mL/h | 153 (94 - 246) | 127.5 (91.2 - 172.4) | < 0.01 |
| Fluid balance |  |  |  |
| Cumulative, mL | 1403 (233 - 2765) | 506 (-1477 - 2269) | < 0.01 |
| Mean hourly, mL/h | 93.4 (21.2 - 183.8) | 21.1 (-61.5 - 94.5) | < 0.01 |
| NAE score |  |  |  |
| Highest | 0.1 (0.0 - 0.4) | 0.1 (0.0 - 0.4) | 0.04 |
| Mean hourly | 0.1 (0.0 - 0.2) | 0.1 (0.0 - 0.2) | 0.01 |
| VI score |  |  |  |
| Highest | 13.3 (0.0 - 33.8) | 12.5 (1.3 - 33.4) | 0.06 |
| Mean hourly | 6.8 (0.0 - 19.6) | 5.4 (0.1 - 17.4) | 0.01 |
| Mean arterial pressure, mmHg |  |  |  |
| Lowest | 66.1 (62.3 - 71.5) | 66.1 (62.1 - 71.1) | < 0.01 |
| Mean hourly | 74.0 (69.9 - 81.0) | 74.8 (70.1 - 83.6) | 0.01 |
| Data are median (quartile 25% - quartile 75%) or No (%). Percentages may not total 100 because of rounding.  Medians reported are with respect to distribution of the values over the entire cohort, whereas the mean hourly values reported refer the values averaged over time for each individual patient.  Abbreviations: CRRT: continuous renal replacement therapy; NAE: noradrenaline equivalent; VI: Vasopressor-inotropic | | | |

| **eTable 2 - CRRT Characteristics During the Treatment Period in the Included Patients** | |
| --- | --- |
|  | **Overall**  **(*n* = 1057)** |
| **During CRRT treatment** |  |
| Duration of treatment, hours | 67 (29 - 169) |
| Net ultrafiltration |  |
| Cumulative, mL/kg | 38.3 (2.9 - 136.0) |
| Mean hourly, mL/kg/h | 0.7 (0.1 - 1.5) |
| Median hourly, mL/kg/h | 0.6 (0.0 - 1.5) |
| Fluid removal |  |
| Cumulative, mL | 3119.0 (232.0 - 11910.0) |
| Mean hourly, mL/h | 66.0 (7.2 - 128.1) |
| Blood flow rate |  |
| Mean hourly, mL/h | 167.3 (150.0 - 195.0) |
| Dialysate rate |  |
| Mean hourly, mL/h | 1000.0 (1000.0 - 1114.2) |
| Post replacement rate |  |
| Mean hourly, mL/h | 684.2 (222.1 - 1000.0) |
| Ultrafiltration flow rate |  |
| Mean hourly, mL/h | 1422.6 (992.6 - 2161.6) |
| Highest, mL/h | 1940.0 (1132.5 - 2991.0) |
| **During follow-up** |  |
| Creatinine, µmol/L |  |
| Highest | 382.0 (249.0 - 521.0) |
| Lowest | 121.0 (78.0 - 177.0) |
| Urea, mmol/L |  |
| Highest | 26.4 (17.4 - 34.7) |
| Lowest | 7.9 (5.5 - 11.4) |
| Potassium, mmol/L |  |
| Highest | 5.4 (4.9 - 6.0) |
| Lowest | 3.4 (3.2 - 3.6) |
| Lactate, mmol/L |  |
| Highest | 3.7 (2.1 - 8.3) |
| Lowest | 0.8 (0.6 - 1.1) |
| Organ support |  |
| Invasive ventilation - no. (%) | 881 (83.6) |
| Vasopressor - no. (%) | 900 (85.1) |
| Days receiving vasopressor | 3.0 (2.0 - 6.0) |
| Data are median (quartile 25% - quartile 75%) or No (%). Percentages may not total 100 because of rounding.  Medians reported are with respect to distribution of the values over the entire cohort, whereas the mean hourly values reported refer the values averaged over time for each individual patient.  Abbreviations: CRRT: continuous renal replacement therapy. | |

| **eTable 3 - Unadjusted Study Outcomes Before and in the 24 Hours After CRRT Commencement for all patients** | | | | |
| --- | --- | --- | --- | --- |
|  | **Pre-CRRT**  **(*n* = 1057)** | **Post-CRRT**  **(*n* = 1057)** | **Mean Difference**  **(95% CI)** | ***p* value** |
| Urine output, mL/h | 60.2 ± 142.8 | 34.7 ± 108.8 | -26.96 (-32.10 to -21.82) | < 0.01 |
| Urine output, mL/kg/h | 0.75 ± 1.78 | 0.43 ± 1.41 | -0.34 (-0.40 to -0.27) | < 0.01 |
| Fluid balance, mL/h | 149.7 ± 328.6 | 20.4 ± 142.1 | -129.33 (-147.12 to -111.53) | < 0.01 |
| Data are median (quartile 25% - quartile 75%) or No (%). Percentages may not total 100 because of rounding.  All data summarised as the mean per patient in the pre-defined period.  CI is confidence interval. | | | | |

| **eTable 4 - Unadjusted Study Outcomes Before and in the 24 Hours After CRRT Commencement Excluding Patients Who Received Frusemide Before or After CRRT** | | | | |
| --- | --- | --- | --- | --- |
|  | **Pre-CRRT**  **(*n* = 786)** | **Post-CRRT**  **(*n* = 786)** | **Mean Difference**  **(95% CI)** | ***p* value** |
| Urine output, mL/h | 58.2 ± 133.0 | 35.1 ± 112.7 | -24.9 (-30.7 to -19.2) | < 0.01 |
| Urine output, mL/kg/h | 0.72 ± 1.75 | 0.43 ± 1.43 | -0.32 (-0.40 to -0.25) | < 0.01 |
| Fluid balance, mL/h | 173.3 ± 370.6 | 38.0 ± 148.0 | -135.24 (-158.5 to -112.0) | < 0.01 |
| Data are median (quartile 25% - quartile 75%) or No (%). Percentages may not total 100 because of rounding.  All data summarised as the mean per patient in the pre-defined period.  CI is confidence interval. | | | | |

**eFigure 1 – Q-Q Plot of Urine Output Data
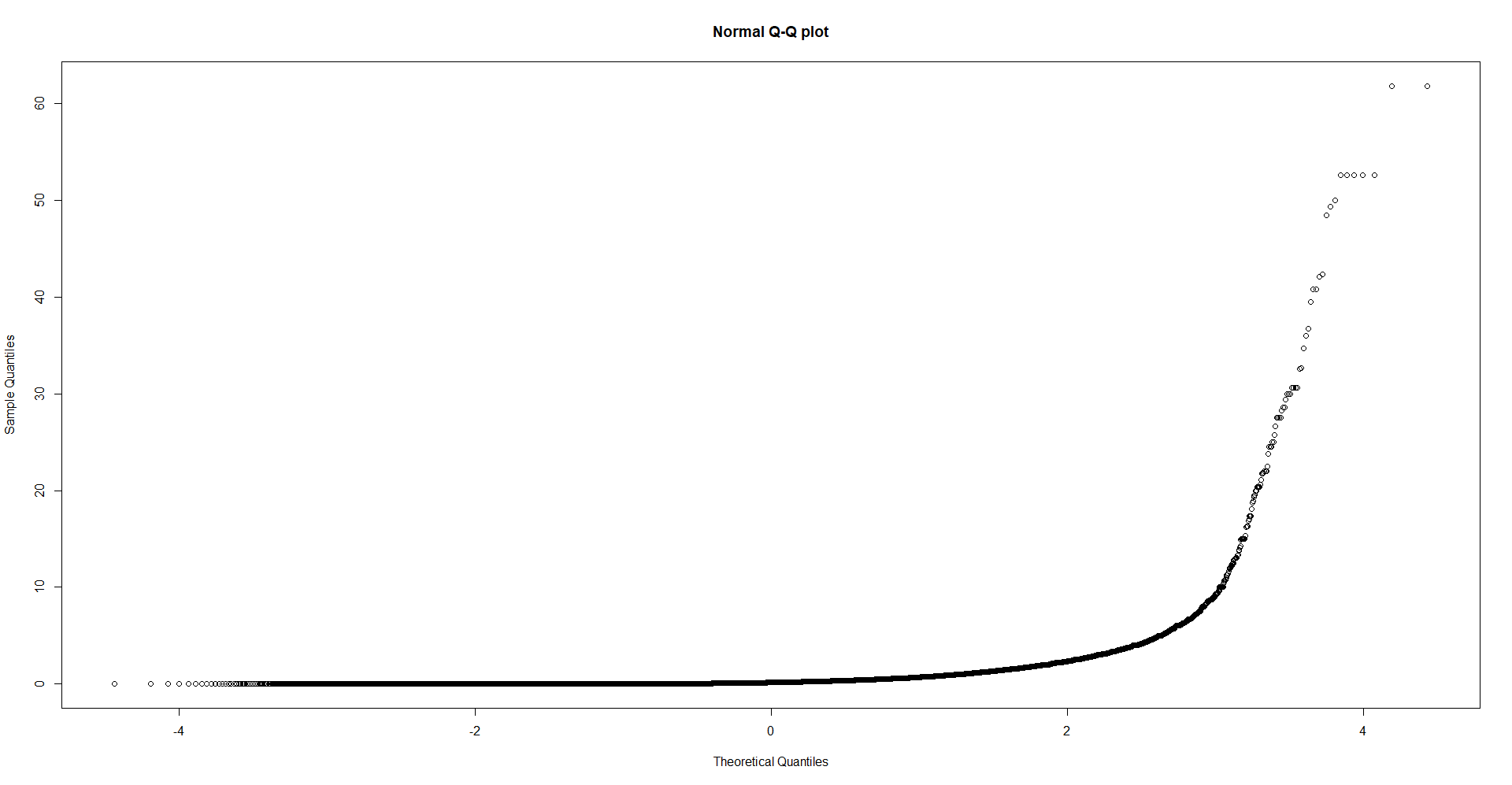
**

**eFigure 2 - Estimates of the Primary and Secondary Outcomes in the Segmented Regression Considering Individual Data**


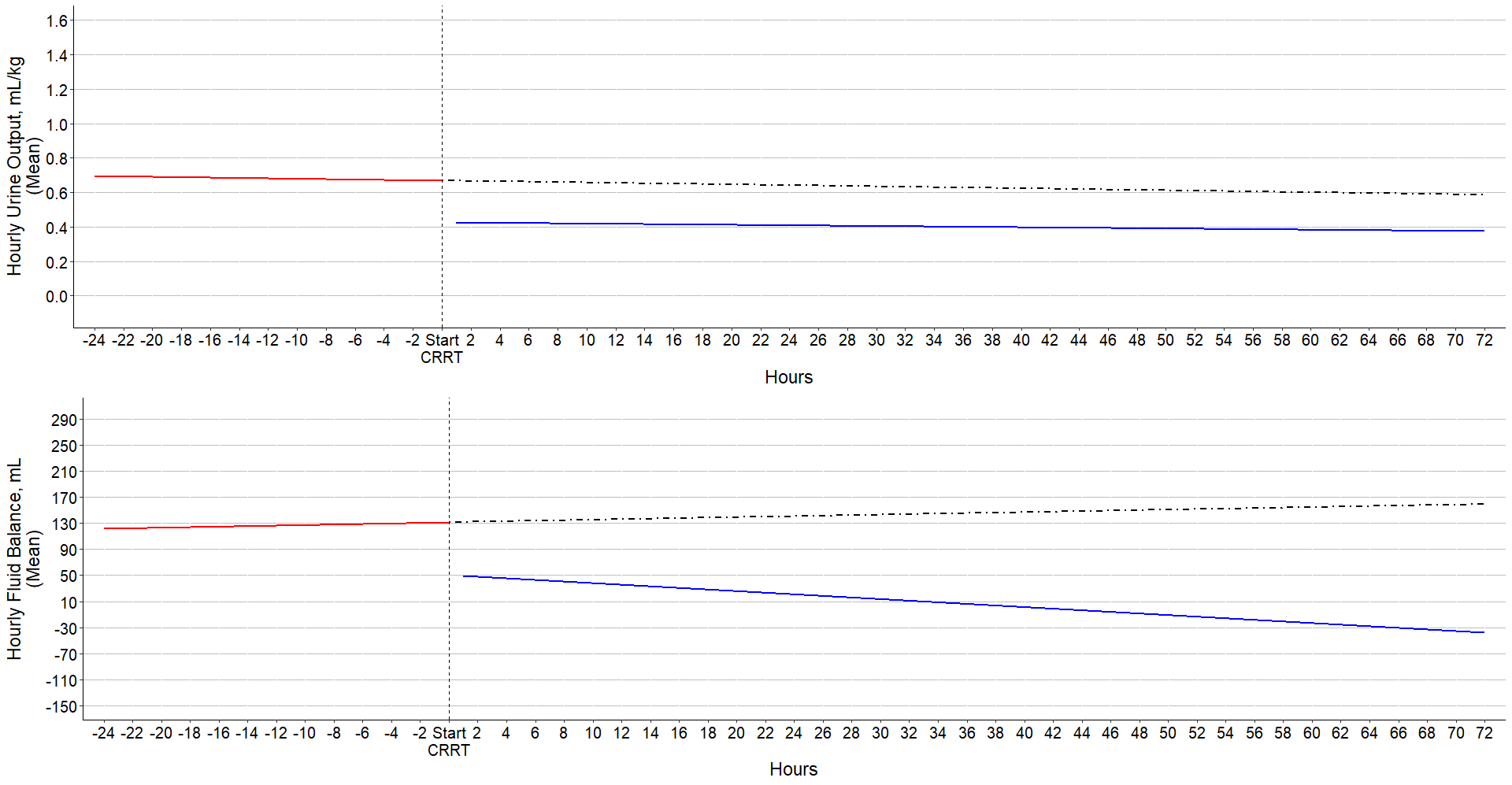


Solid (blue) line represents fitted line from the segmented regression and dashed line is the counterfactual outcome had the pre-CRRT trends continued. Individual values were omitted for clarity. The solid (blue) line below the black dotted line indicates that adjusted values for the post-CRRT period are lower than the counterfactual.

## **eFigure 3 - XY Plot of Change in Urine Output versus Change in Fluid Balance in Non-oliguric patients only**


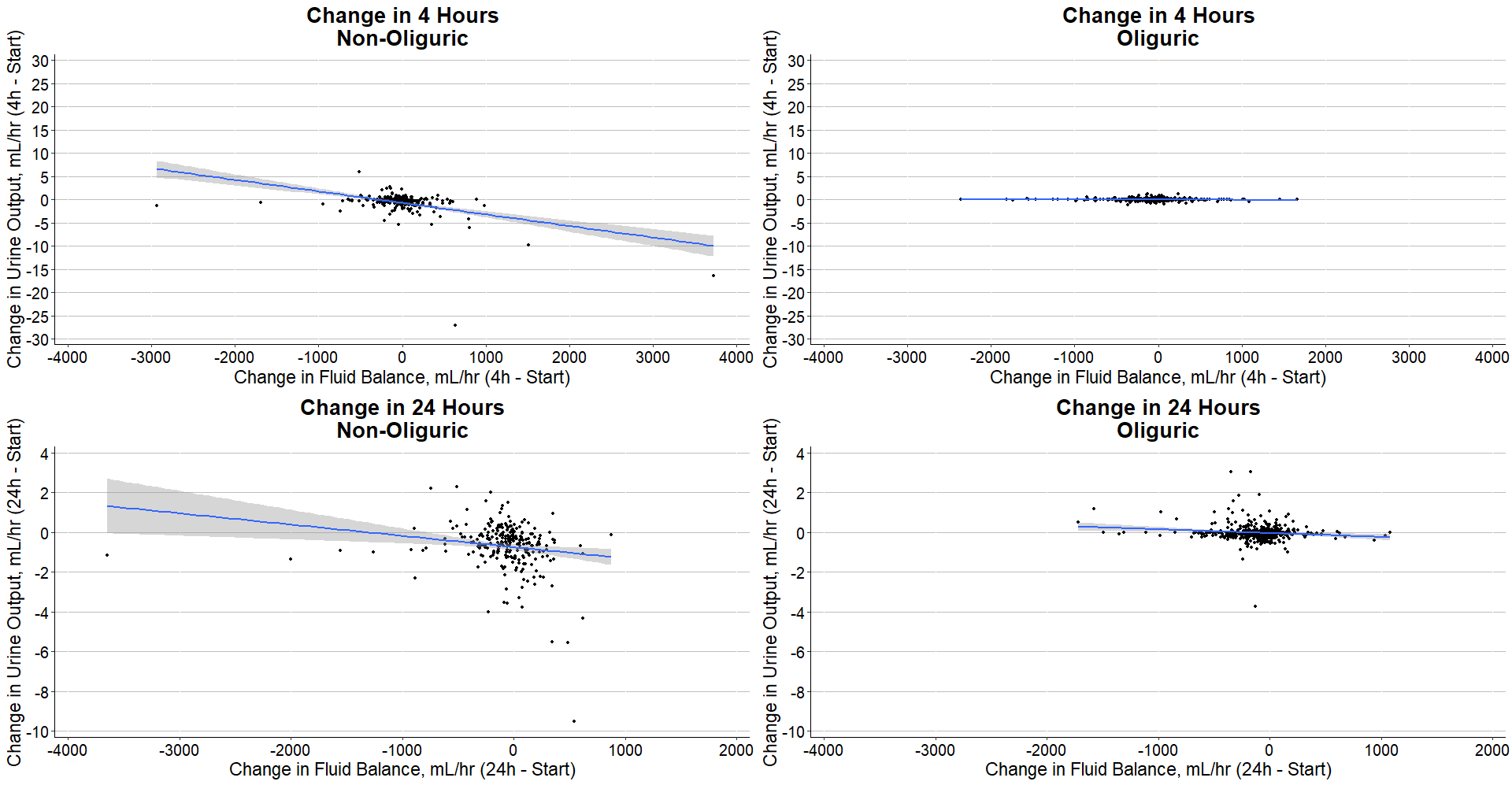

Supplement: Supplementary file 1 [file Supplementary_material-Suppl.1-s1.docx]
